# The molecular chaperone Hsp70 promotes the proteolytic removal of oxidatively damaged proteins by the proteasome

**DOI:** 10.1016/j.freeradbiomed.2016.08.002

**Published:** 2016-08-03

**Authors:** Sandra Reeg, Tobias Jung, José P. Castro, Kelvin J.A. Davies, Andrea Henze, Tilman Grune

**Affiliations:** a German Institute of Human Nutrition Potsdam Rehbruecke (DIfE), Department of Molecular Toxicology, 14588 Nuthetal, Germany; b German Center for Diabetes Research (DZD), Germany; c University of Southern California, Leonard Davis School of Gerontology, and Division of Molecular & Computational Biology, Dornsife College of Letters, Arts, and Sciences, Los Angeles, CA 90089-0191, USA; d University Potsdam, Institute of Nutritional Science, Department of Physiology and Pathophysiology, 14588 Nuthetal, Germany

**Keywords:** Protein oxidation, Proteasome, Chaperone, HSP70

## Abstract

One hallmark of aging is the accumulation of protein aggregates, promoted by the unfolding of oxidized proteins. Unraveling the mechanism by which oxidized proteins are degraded may provide a basis to delay the early onset of features, such as protein aggregate formation, that contribute to the aging phenotype. In order to prevent aggregation of oxidized proteins, cells recur to the 20S proteasome, an efficient turnover proteolysis complex. It has previously been shown that upon oxidative stress the 26S proteasome, another form, dissociates into the 20S form. A critical player implicated in its dissociation is the Heat Shock Protein 70 (Hsp70), which promotes an increase in free 20S proteasome and, therefore, an increased capability to degrade oxidized proteins. The aim of this study was to test whether or not Hsp70 is involved in cooperating with the 20S proteasome for a selective degradation of oxidatively damaged proteins. Our results demonstrate that Hsp70 expression is induced in HT22 cells as a result of mild oxidative stress conditions. Furthermore, Hsp70 prevents the accumulation of oxidized proteins and directly promotes their degradation by the 20S proteasome. In contrast the expression of the Heat shock cognate protein 70 (Hsc70) was not changed in recovery after oxidative stress and Hsc70 has no influence on the removal of oxidatively damaged proteins. We were able to demonstrate in HT22 cells, in brain homogenates from 129/SV mice and *in vitro*, that there is an increased interaction of Hsp70 with oxidized proteins, but also with the 20S proteasome, indicating a role of Hsp70 in mediating the interaction of oxidized proteins with the 20S proteasome. Thus, our data clearly implicate an involvement of Hsp70 oxidatively damaged protein degradation by the 20S proteasome.

## 1. Introduction

Members of the 70 kDa-heat shock protein (Hsp70) family are, in their function as molecular chaperones, involved in folding of newly synthesized proteins and refolding of damaged or misfolded proteins, as well as in assembly and disassembly of protein complexes. All human Hsp70 family members have highly conserved domain structures [1]. They consist of an N-terminal ATPase domain, a middle region and a C-terminal peptide binding domain. However, they differ in expression patterns, cellular localization and function. There are Hsp70 family members specifically located in the endoplasmatic reticulum (Grp78, also known as BiP) and in the mitochondria (Grp75, also known as mortalin). However, the members which are mainly located in the cytosol and nucleus are the heat shock cognate protein 70 (Hsc70, also known as Hsp73) and the heat shock protein 70 (Hsp70, also known as Hsp72).

Hsc70 is a constitutively expressed member of the Hsp70 family. It is mainly involved in folding of newly synthesized polypeptides to nascent proteins, protein translocation and disassembly of clathrin-coated vesicles. Hsp70 shows 86% structure similarity to Hsc70 [1], but in contrast to Hsc70, the expression of Hsp70 is induced by various stressors, such as oxidative stress. Cellular stress often leads to protein unfolding and, therefore, to increased protein hydrophobicity, which may result in the formation of toxic protein aggregates [2,3]. However, the increased protein hydrophobicity leads to the activation of a highly regulated and rapid series of events, called the ‘heat shock response’, resulting in the fast expression of heat shock proteins, such as Hsp70 (for review see [4]). An unfolded protein that binds to Hsp70 may be either refolded into its native conformation and then released, or may stay bound by Hsp70 to protect non-damaged molecules from unspecific aggregation. Since most oxidative protein modifications are not repairable, the majority of oxidized proteins have to be degraded by the proteasomal system in the cytoplasm, nucleus and endoplasmic reticulum.

The two major forms of the proteasomal system are the 20S proteasome and the 26S proteasome. The 20S proteasome is a 700 kDa barrel-shaped multi-enzyme complex, composed of four homologous rings. The inner rings consist of seven different β-subunits (β1–β7) and they display the catalytic center of the 20S proteasome, since three of these subunits possess (different) proteolytic activities. The two outer rings are composed of seven different α-subunits (α1–α7). They form the 20S proteasome gate and regulate recognition and access of the substrates to the catalytic center. The 26S proteasome consists of the 20S core and one or two bound 19S regulators that conduct recognition and binding of polyubiquitinated protein substrates, ATP-dependent ubiquitin removal and substrate unfolding.

It has been extensively documented that oxidized proteins are excellent substrates for the 20S proteasome, which in contrast to the 26S proteasome, is able to degrade unfolded proteins in an ATP- and ubiquitin-independent fashion [5–9]. The 26S proteasome catalyzes the degradation of proteins with a more or less intact three-dimensional structure, such as regulatory proteins that are no longer needed in cellular metabolism, and partially misfolded proteins resulting from errors in protein synthesis [10–12].

Hsp70 seems to have a key role in the disassembly of the 26S proteasome into 20S proteasome and 19S regulators during oxidative stress, leading to an increased quantity of uncapped 20S proteasome particles and, therefore, to an elevated capacity to degrade oxidized proteins [13]. Interestingly, it was shown that Hsp70 and Hsc70 are directly involved in recruiting substrate proteins for different degradation processes, such as chaperone-mediated autophagy and aggrephagy [14–16], as well as for degradation *via* the ubiquitin-(26S) proteasome-system [17–20]. Regarding the degradation of oxidized proteins, it was previously observed that another important heat shock protein Hsp90 is involved in preserving the integrity and stability of the 20S proteasome during oxidative stress [21] and it also seems to promote the degradation of oxidatively damaged proteins [22].

Since no direct involvement of Hsp70 chaperones in the selective proteolytic removal of oxidized proteins has previously been reported, we hypothesized on the role of either Hsp70 or Hsc70 in the assistance of oxidized proteins degradation by the 20S proteasome. We were able to clearly demonstrate *in vitro*, in HT22 cells and in mice that Hsp70 directly interacts with both, oxidized proteins and the 20S proteasome, and directly promotes the rapid and selective proteolytic removal of oxidatively damaged proteins.

## 2. Materials and methods

### 2.1. Cell culture and treatment

Experiments were performed using HT22 cells, a mouse hippocampal neuronal cell line. HT22 cells were cultured in Dulbecco’s modified Eagle’s medium (DMEM) supplemented with 10% fetal calf serum, 1% l-alanyl-l-glutamine and 0.35% additional glucose, in an atmosphere of 7% CO_2_ and 37 °C.

HT22 cells were treated with hydrogen peroxide (H_2_O_2_) (Sigma Aldrich, Taufkirchen Germany) at various concentrations (diluted in PBS), and incubated for 0.5 hr at 37 °C. Control cells were incubated in PBS for the same time. After oxidant treatment, cells were cultured in fresh growth medium for various periods of recovery. To inhibit the activity of the proteasome, the proteasomal inhibitor lactacystin (LC) (Sigma Aldrich, Taufkirchen, Germany) was used. Cells were pre-incubated for 2 hr with 2 µM LC. After hydrogen peroxide/PBS-treatment, 2 µM LC was again added to the growth medium. For heat shock conditions, petri dishes were tightly wrapped with parafilm and incubated in a water bath at 42 °C for 1h. After that petri dishes were kept in a CO_2_ incubator at 37 °C for the times indicated.

### 2.2. Cell viability

Cell viability was measured using the 3-(4,5 Dimethylthiazol-2-yl)-2,5-diphenyltetrazolium bromide (MTT) viability assay. MTT was dissolved in culture medium (5 mg/ml) and added to the cells. NAD(P)HR-dependent oxidoreductases reduce MTT to its insoluble formazan. After an incubation of 1 hr, MTT solution was discarded and cells were solubilized by adding DMSO containing 10% SDS and 0.6% acetic acid. The absorption of the formazan was measured at a wavelength of 580 nm and the intensity correlates with the quantity of living cells.

### 2.3. Mice

Male SV129 mice (18 month) were bred *in house* in the animal facility of the German Institute of Human Nutrition (Nuthetal, Germany). Mice were reared under SPF-conditions and housed in groups (3–5 animals per cage) under standard laboratory housing conditions with a diurnal 12 hr light and dark cycle. Animals had free access to fresh tap water and food (standard diet R/M-H, Ssniff, Soest, Germany). All animal experiments were performed in compliance with the German animal protection law (TierSchG). Mice were housed and handled in accordance with good animal practice as defined by FELASA (www.felasa.eu/guidelines.php) and the national animal welfare body GV-SOLAS (www.gv-solas.de/index.html). The local authorities approved all animal experiments (LAVG Brandenburg, Germany; Permit-Number: T-01-15-MTOX).

### 2.4. Immunoblot analysis

HT22 cells were lysed using a SDS lysis buffer (10 mM TrisHCl, pH 7.5, 0.9% Nonidet P-40, 0.1% SDS and 1 mM Pefabloc) and repeated disruption steps by a 27 G-needle. Cell lysates were centrifuged at 20,000 × *g* for 10 min. The protein content of supernatants was measured by DC protein assay (BioRad, Munich, Germany). Reducing Laemmli buffer (0.25 M Tris, pH 6.8, 8% SDS, 40% glycerol, 0.03% orange G) was added to 20 µg – 30 µg protein of each sample. Protein was denatured at 95 °C for 5 min. Afterwards, samples were separated by SDS-PAGE and transferred to a nitrocellulose membrane. Blocking was performed using Odyssey^®^ Blocking Buffer (LI-COR, Bad Homburg, Germany) 1:2 diluted in PBS. Primary and secondary antibodies were also diluted in Odyssey^®^ Blocking Buffer/PBS, containing 0.1% Tween-20. Following primary antibodies were used: Hsp70/Hsp72 antibody [C92F3A] (ADI-SPA-810, Enzo Life Sciences, Loerrach, Germany), Hsp701A Polyclonal antibody (PA5–28003, Thermo Fisher Scientific, Schwerte, Germany), Anti-Hsc70 antibody [13D3] (ab2788, Abcam, Cambridge, UK), Proteasome 20S core subunits, pAb (BML-PW8155, Enzo Life Sciences, Loerrach, Germany), proteasomal subunit α4 (PSMA4) (BML-PI104, Enzo Life Sciences, Loerrach, Germany), Enzo Life Sciences, Loerrach, Germany), Anti-GAPDH [6C5] antibody (ab8245, Abcam, Cambridge, UK), mono- and polyubiquitinated conjugates [FK2] (BML-PW8810, Enzo Life Sciences, Loerrach, Germany), IRDye^®^ secondary antibodies (LI-COR, Bad Homburg, Germany) were used for detection of immunoblots with the Odyssey^®^ imaging system (LI-COR, Bad Homburg, Germany).

#### 2.4.1. Protein carbonyl immunoblot

To detect protein carbonyls, as a common biomarker for protein oxidation, membrane-bound protein carbonyls were derivatized by 2,4-dinitrophenylhydrazine, described in [23], resulting in the formation of dinitrophenyl (DNP) hydrazone products [24] which are detected by anti-DNP antibody (D9656, Sigma-Aldrich, Taufkirchen, Germany). The intensity of the whole protein carbonyl lane was quantified and used for calculation.

#### 2.4.2. Hsp70 and Hsc70 separation

In contrast to the separation of Hsp70 and Hsc70 from heat-shocked cells (with a very high induction of Hsp70), where 10% gels and GAPDH as the loading control were used, Hsp70 (72 kDa) and Hsc70 (73 kDa) from oxidant-treated cells (with low amounts of Hsp70 in comparison to Hsc70) were separated using 7.5% gel with an elution of all proteins up to 60 kDa to improve their separation. Therefore, detection of the loading control GAPDH was not possible and a staining of membrane-bound proteins was performed using Ponceau solution (containing 0.2% Ponceau S and 1% acetic acid).

### 2.5. Hsp70 knockdown and Hsp70-GFP overexpression

To knock down Hsp70 and Hsc70 expression, and prevent induction during treatments, RNA interference experiments were performed using ON-TARGET plus SMARTpool siRNAs (GE Healthcare/Dharmacon, Freiburg, Germany). Cells are cultured in antibiotic-free medium. Then, 24h after seeding, cells were transfected with siRNA and DharmaFECT 3 Transfection Reagent (GE Healthcare/Dharmacon, Freiburg, Germany) according to the manufacturer's instructions. For Hsp70 knockdown 12.5 nM Hspa1a siRNA and 12.5 nM Hspa1b siRNA were used. Hsc70 knockdown was performed using 6.25 nM Hspa8 siRNA. Furthermore, non-targeting scrambled siRNA was used as negative control. Appropriate siRNA concentrations were tested before on cell viability using MTT assay (Fig. 3B).

To express an Hsp70-GFP fusion protein, the cells were transfected using TurboFect Transfection Reagent (Thermo Fisher Scientific, Schwerte, Germany) 24h after seeding. The vector was purchased from Origene (Rockville, USA): pCMV6-AC-GFP with Insert HSPA1A (human heat shock 70 kDa protein 1A) (RG200270, Origene, Rockville, USA). A pCMV6-AC-GFP without Insert (PS100010, Origene, Rockville, USA) transfected with the same conditions was used as control.

### 2.6. Proteasomal activity against a fluorogenic peptide substrate

HT22 cells were lysed using a lysis buffer, containing 250 mM sucrose, 25 mM Hepes, 10 mM magnesium chloride, 1 mM EDTA and 1.7 mM DTT, and repeated freeze thaw cycles. Cell lysates were centrifuged at 18,000 × *g* for 10 min. The protein content of the supernatant was measured with the Bradford assay (BioRad, Munich, Germany). For the measurement of 20S proteasome activity ATP was depleted using 0.1 mg/ml hexokinase and 15 mM 2-deoxyglucose. To distinguish the ATP-stimulable 26S proteasome activity, ATP to a final concentration of 2 mM was added in another experimental setup. Basal buffer was a 225 mM Tris buffer, pH 7.8, supplemented with 45 mM potassium chloride, 7.5 mM magnesium acetate, 7.5 mM magnesium chloride and 1 mM DTT. Chymotrypsin-like proteasomal activity was measured using fluorogenic peptide suc-Leu-Leu-Val-Tyr-7-amino-4-methylcoumarin (Sigma Aldrich, Taufkirchen, Germany). Protected from light, samples were incubated for 30 min at 37 °C. Proteasomal degradation of the fluorogenic peptide leads to the liberation of the fluorescent product 7-amino-4-methylcoumarin (AMC). The content of AMC liberation was measured with the plate reader at 360 nm excitation and 460 nm emission. As standards, free AMC in various concentrations was used.

### 2.7. Proteasomal degradation of oxidized actin

5 µM of non-muscle actin (APHL99, Tebu-Bio, Offenbach, Germany) were incubated in a 1:2 ratio with ‘General Actin Buffer’ (GAB) (BSA01, Tebu-Bio, Offenbach, Germany) containing 5% sucrose, 1% dextran and 0.2 mM ATP for 1 hr at 4 °C. For oxidation, actin was incubated with 0.1 mM hydrogen peroxide, diluted in GAB for 2h at 25 °C. To stop the reaction, 40 µg/ml catalase and 50 mM DTT were added to the mixture. Afterwards, actin was incubated with 15 nM isolated human 20S proteasome (described in [25]) and 300 nM human recombinant Hsp70 (Enzo Life Sciences, ADI-ESP-555, Loerrach, Germany) for 2 hr at 37 °C. Since, fluorescamine reacts with free amino groups, leading to the formation of a fluorescent product, fluorescamine assay was performed as described in [26] to calculate the proteasomal degradation of actin. Dilutions of glycine were used as standards to calculate the amount of free amino groups. Proteasomal degradation was calculated as the difference between the sample with 20S proteasome and without 20S proteasome to exclude the 20S proteasome-independent formation of free amines during oxidation.

### 2.8. Proteolytic degradation assay

Proteolytic degradation was measured in HT22 cells as described in [27] with small changes. To label short-lived cellular proteins, cells were incubated for 2 hr in DMEM without methionine/cysteine (Sigma Aldrich, Taufkirchen, Germany) supplemented with EXPRE35S35S Protein Labeling Mix [^35^S] (Perkin Elmer, NEG072007MC, Baesweiler, Germany) to a final concentration of 100 µCi [^35^S]methionine/cysteine per ml. After removing the labeling growth medium, cells were incubated for various time points in normal growth medium supplemented with 10 mM methionine/cysteine. Measurement of proteolytic degradation was performed as described in [27].

### 2.9. Interaction analysis of Hsp70, oxidized proteins and 20 S proteasome

#### 2.9.1. Immunoprecipitation of Hsp70 from HT22 cell lysates

Immunoprecipitation of Hsp70 was performed with an Immunoprecipitation Kit Dynabeads^®^ Protein G (Thermo Fisher Scientific, Schwerte, Germany) as described in the manufacturer's protocol. HT22 cells were lysed in PBS due to repeated disruption steps and 10 min centrifugation at 20,000 × *g*. Hsp70 was eluted using the mouse monoclonal Hsp70/Hsp72 antibody [C92F3A] (ADI-SPA-810, Enzo Life Sciences, Loerrach, Germany). Unbound fractions and immunoprecipitate were analyzed using protein carbonyl immunoblot. Hsp70 was detected using polyclonal rabbit Hsp701A antibody (PA5-28003, Thermo Fisher Scientific, Schwerte, Germany), which detects also Hsc70. To exclude that also Hsc70 is immunoprecipitated we detected also Hsc70 using the monoclonal Hsc70 antibody (ab2788, Abcam, Cambridge, UK).

To detect the interaction of Hsp70 with proteasomal subunits the Pierce Co-Immunoprecipitation kit (Thermo Fisher Scientific, Schwerte, Germany) was used, which allows no co-elution of antibody heavy and small chains, since the latter interferes in the analysis of proteasomal subunits in immunoblot detection. Hsp70 was detected using the mouse monoclonal Hsp70/Hsp72 antibody [C92F3A].

#### 2.9.2. Immunoprecipitation of Hsp70 from murine brain lysates

Mice were sacrificed by cervical dislocation. Immediately after harvesting, the brains were placed on ice and homogenization of the brains was performed in PBS with a Potter-Elvehjem tissue homogenizer. The immunoprecipitation of Hsp70 was performed using the Pierce Co-Immunoprecipitation kit (Thermo Fisher Scientific, Schwerte, Germany) and the mouse monoclonal Hsp70/Hsp72 antibody [C92F3A] (ADI-SPA-810, Enzo Life Sciences, Loerrach, Germany). The immunoprecipitate was analyzed performing an immunoblot for protein carbonyls, proteasomal subunits and Hsp70. Hsp70 was detected with the mouse monoclonal Hsp70/Hsp72 antibody [C92F3A].

#### 2.9.3. Density gradient centrifugation with co-elution of Hsp70 in purified proteasome fractions

HT22 cells were transfected with HSPA1A-AC-GFP expression vector. 24 hr after transfection, cells were treated with 0.5 mM hydrogen peroxide. After a recovery phase of 2 hr and 8 hr cells were lysed with PBS. Separation of proteasomal fractions was performed using density gradient centrifugation. Lysates were added to a 10–40% sucrose gradient, prepared with a buffer, containing 1 mM Tris, 25 mM KCl and 5 mM NaCl. Centrifugation was performed at 72,000 × *g* for 34 hr at 4 °C. After centrifugation, the gradient was separated into fractions of 0.5 ml. Proteasomal activity of the fractions was analyzed. Fractions, high in proteasomal activity were pooled and analyzed *via* immunoblot. For detection of the Hsp70-GFP fusion protein Hsp701A Polyclonal antibody (PA5-28003, Thermo Fisher Scientific, Schwerte, Germany) was used. The content of Hsp70 was related to the content of 20S proteasome in the pooled proteasome fractions.

#### 2.9.4. In vitro approach to analyze the interaction of Hsp70 with oxidized actin

To investigate the interaction between Hsp70 and 20S proteasome during oxidative stress and protein oxidation *in vitro*, the experimental approach was performed as described before in ‘Proteasomal degradation of oxidized actin’ with the following small changes. After the actin oxidation procedure, 20S proteasome and Hsp70, which was previously incubated with 5 mM of the heterobifunctional cross-linker Sulfo-NHS-LC-Diazirine (sulfosuccinimidyl 6-(4,4′-azipentanamido)hexanoate) (Sulfo-LC-SDA) (26,174, Thermo Fisher Scientific, 26,174, Schwerte, Germany) were added to the mixture and incubated for 30 min at 37 °C. The cross-linker was activated by 15 min of UVA illumination, as described in the manufacturer's protocol. Afterwards, the mixture was incubated for further 1.5 hr at 37 °C. Reactions were quenched by addition of Tris at a final concentration of 50 mM. *Via* SDS-PAGE, proteins and possible interaction products were separated and analyzed using MALDI-TOF mass spectrometry. The amount of Hsp70 and actin in the interaction bands was checked by immunoblot analysis.

### 2.10. MALDI-TOF mass spectrometry and protein identification

Protein complexes were separated by SDS-PAGE as previously described and gels were stained by colloidal Coomassie brilliant blue staining according to Candiano et al. [28]. Protein bands were excised from gel, destained, reduced with TCEP, alkylated with iodacetamide and subsequently digested with trypsin overnight. The reaction was quenched by the addition of 1% TFA. Digests were desalted and cleaned by ZipTips^®^ C_18_ (Merck Millipore, Darmstadt, Germany) according to manufacturer's instructions. Peptides were eluted from ZipTips^®^ with HCCA (50% in 50% acetonitrile and 0.5% TFA (Bruker, Germany)) and 1 µl of the eluent was applied to a polished steel target (Bruker, Germany).

Tryptic peptides were analyzed with an AutoflexSpeed MALDI-TOF mass spectrometer (Bruker, Karlsruhe, Germany). The analyses were performed in positive ion and reflector TOF mode with 19 kV and 21 kV acceleration voltage, respectively, using a peptide mixture for external calibration (Bruker, Karlsruhe, Germany). Peak lists were generated using flexAnalysis software (Bruker, Karlsruhe, Germany) and transferred to BioTools software (Bruker, Karlsruhe, Germany) for alignment with SwissProt database *via* the Mascot search engine (www.matrixscience.com). Search parameters were as follows: taxonomy was restricted to primates, MS tolerance was 200 ppm, used enzyme was trypsin, missing cleavages ≤ 1 were accepted, variable modifications were defined as carbamidomethylation of cysteine. For reliable identification with Mascot probability values of <0.05 (corresponding to a protein score >57) were expected. Additionally, results were accepted as potent candidates if sequence coverage was >20%. Furthermore, based on the assumption that cross-linking resulted in the formation of protein complexes that cannot be separated by SDS-PAGE and therefore may hamper the data evaluation, the database alignment was performed with up to three iterations per sample including all unmatched peaks for every following iteration. Only those results were considered that could be confirmed in at least three of five replicates.

### 2.11. Measuring the mobility of Hsp70 using “Fluorescence Recovery After Photobleaching” (FRAP)

For confocal microscopy, HT22 cells were seeded on glass bottom dishes (MatTek, Ashland, USA). 24 hr after seeding, cells were transfected using HSPA1A-AC-GFP expression vector. FRAP measurement was performed using Zeiss Laser Scanning Microscope 780 with Zen10 software. During measurement glass bottom dishes were localized in the incubator at 37 °C and an atmosphere of 7% CO_2_. At the beginning of each bleaching experiment three ROIs (region of interest) with a size of 3 µm^2^ were fixed: ROI^1^ = bleached region in the cytosol; ROI^2^ = control region (region in a cell which is not bleached), ROI^3^ = background region (region with no fluorescence). 50–80% of the fluorescence of Hsp70-GFP fusion protein in ROI^1^ was bleached using following conditions: 100% laser intensity (488 nm), 80 iterations. The recovery time after photobleaching t_1/2_ was calculated with the Zen10 software. For each sample 15–30 cells were measured.

### 2.12. Statistical analysis

Statistical analyses were performed using the Prism 5 software (Graph-Pad, La Jolla, CA, USA). All experiments were carried out at least in independent triplicates and are presented as mean values ± SD. Student's *t*-test was used to compare two groups. To compare more than two groups ordinary one-way ANOVA with Tukey post-hoc test was used. *P* values of <0.05 were accepted as statistically significant differences.

## 3. Results

### 3.1. Hsp70 expression is induced following oxidative stress

**Fig. 1:**
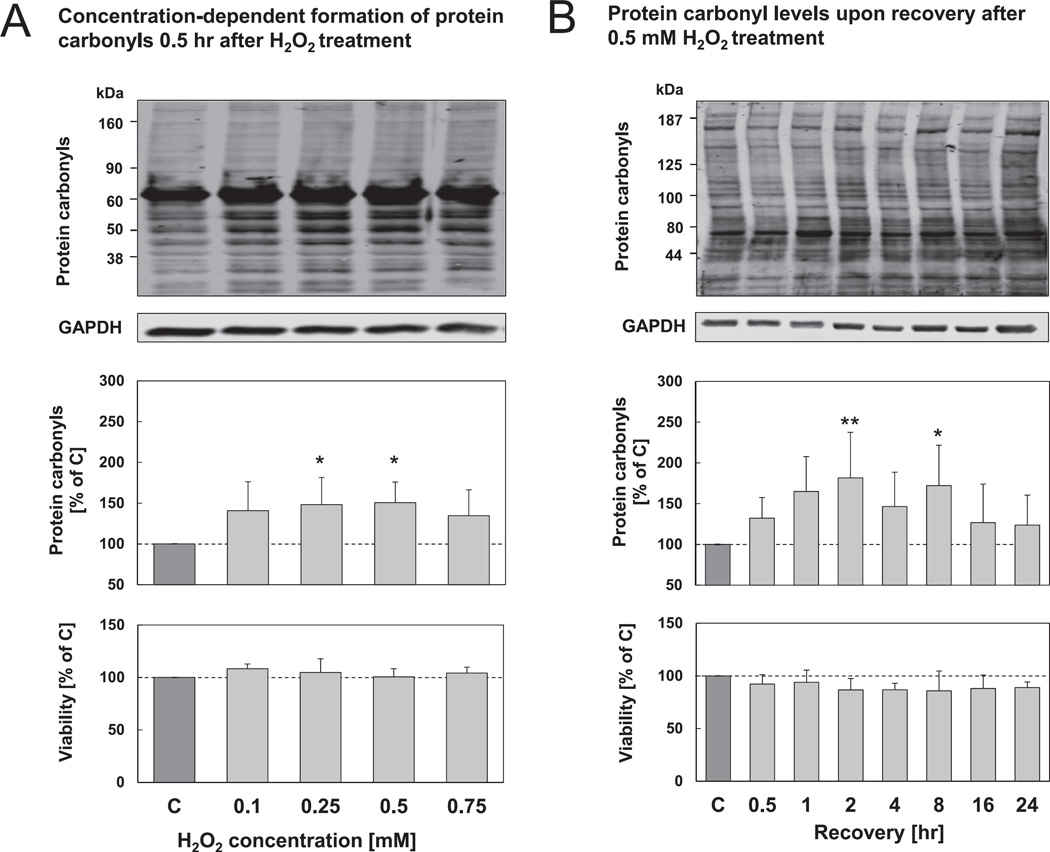
Protein carbonyl levels and viability of HT22 cells after hydrogen peroxide treatment. (A) HT22 cells were treated with various concentrations of hydrogen peroxide for 0.5 hr, control cells were simultaneously incubated with PBS. After treatment, cells were incubated for 0.5 hr in growth medium at 37 °C. (B) HT22 cells were treated with 0.5 mM hydrogen peroxide/PBS for 0.5 hr. Afterwards, cells were incubated for various time periods in growth medium. A, B) Cells were either lysed and used for protein carbonyl immunoblot or used for MTT assay (see methods). The amount of protein carbonyls (whole molecular weight range) was normalized to the density of the GAPDH immunoblot. Representative immunoblots of protein carbonyls and GAPDH are shown. The columns are the means ± SD, n = 5–10, *P < 0.05 *vs*. control, **P < 0.01 *vs*. control.

In order to establish conditions in HT22 cells where protein oxidation is increased but cellular viability is only moderately affected, we tested different concentrations and time points for hydrogen peroxide treatment. To this end, HT22 cells were incubated with varying hydrogen peroxide concentrations for 0.5 hr and analyzed for the formation of oxidized proteins by quantifying protein carbonyls (Fig. 1A). Protein carbonyl levels are a commonly used biomarker to assess the amount of oxidized proteins in cells [24]. As it can be seen, protein carbonyl levels are increased after treatment with 0.25–0.5 mM hydrogen peroxide (Fig. 1A) without affecting any cellular viability. Moreover, protein carbonyl levels were confirmed by performing protein carbonyl ELISA (data not shown). Subsequently we decided to perform further experiments using 0.5 mM hydrogen peroxide and to check the formation of oxidized proteins at various time points (Fig. 1B). We found a significant increase in protein carbonyl levels up to 8 hr after treatment, also without diminishing cell viability. At later time points protein carbonyl levels declined towards basal levels.

**Fig. 2:**
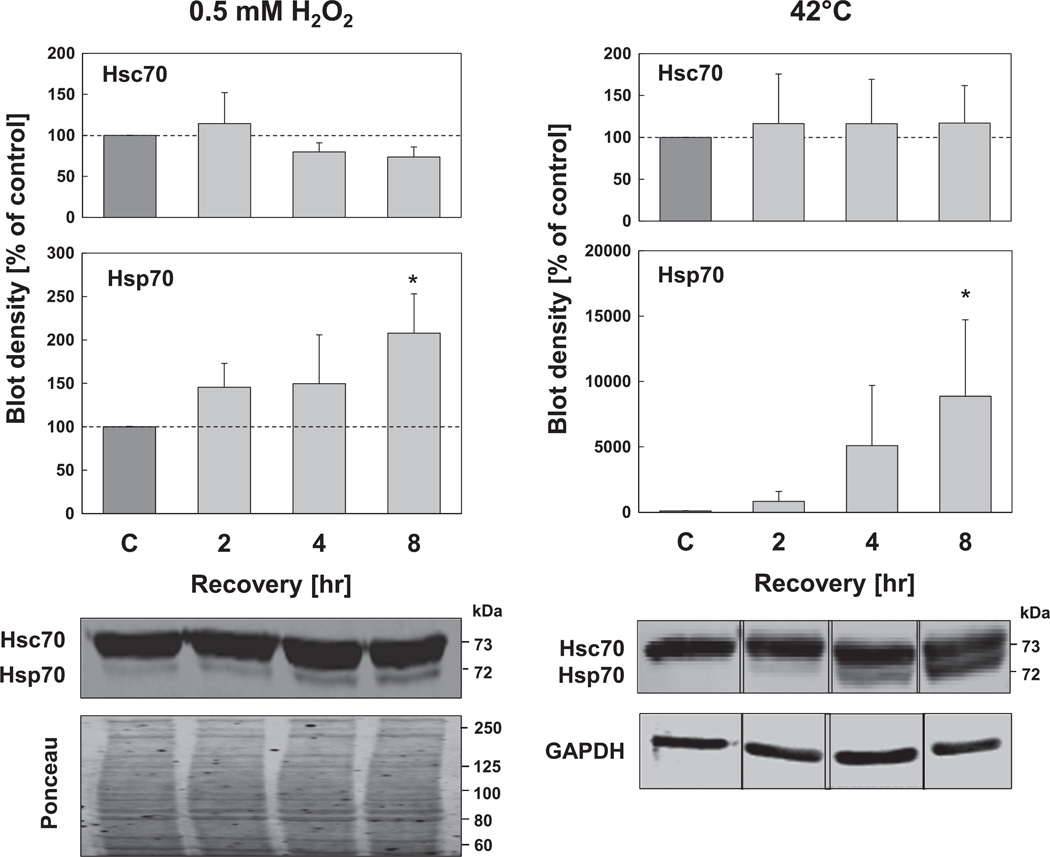
Inducibility of Hsp70 after oxidative stress and heat shock. HT22 cells were either treated with 0.5 mM hydrogen peroxide for 0.5 hr or incubated for 1 hr at 42 °C. At the indicated time points after treatment, cells were lysed and immunoblots were performed. Representative immunoblots of Hsp70 and Hsc70 are shown. Hsp70 and Hsc70 concentrations were normalized to the density of GAPDH immunoblot or Ponceau staining. Columns are the means ± SD, n = 3–4, *P < 0.05 *vs*. control.

In the next experiment we tested Hsp70 and Hsc70 expression levels using the conditions presented in Fig. 1 (0.5 mM hydrogen peroxide for 0.5 hr) to induce mild oxidative stress. Since it is known that chaperone expression patterns vary under different cellular stress conditions, we performed additional experiments using a thermal exposure to 42 °C for 1 hr, as a positive control. As shown in Fig. 2, Hsc70 expression did not change, neither under heat shock, nor under oxidative stress conditions. However, Hsc70 levels were already high, even in non-stressed conditions. In contrast, the expression of Hsp70, known to be a highly inducible protein, was very low under non-stressed conditions and increased by almost 150% 2 hr after oxidative stress initiation, and by more than 200% 8 hr after oxidative stress, compared with control cells (Fig. 2). However, it may also be noted that Hsp70 expression under hyperthermia was more pronounced.

### 3.2. Hsp70 knockdown results in increased accumulation of oxidized proteins

After establishing experimental conditions of mild oxidative stress, where cells are still viable yet protein oxidation is increased and Hsp70 is induced, we decided to use these conditions to test the role of Hsp70 and Hsc70 in the degradation of oxidized proteins. Therefore, we knocked down the expression of both proteins *via* siRNA treatment which should also prevent stress-induced induction.

**Fig. 3:**
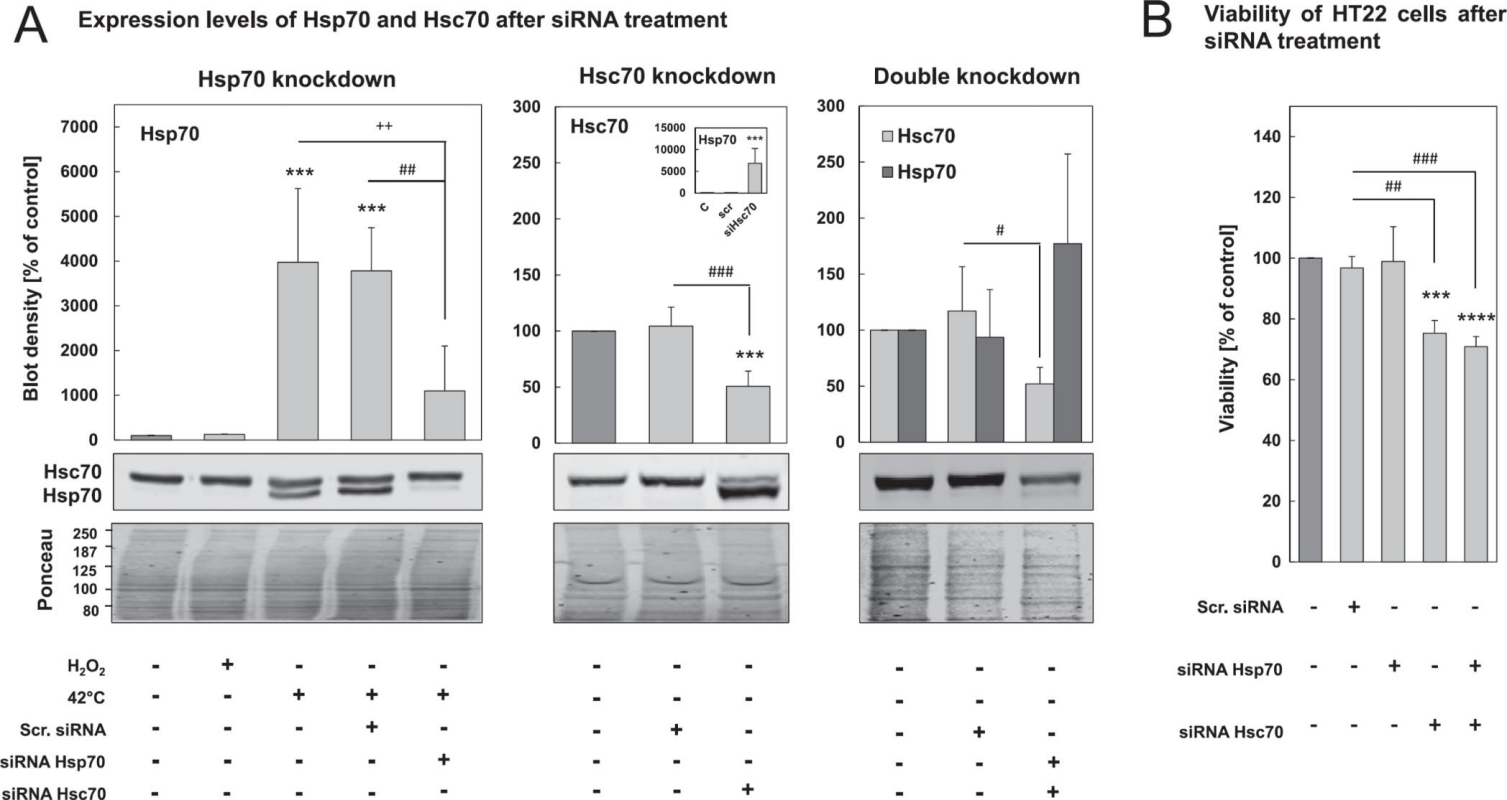
Efficiency of Hsp70 and Hsc70 knockdown. HT22 cells were cultured in antibiotic-free medium. 24 hr after seeding, cells were transfected with 25 nM Hsp70 siRNA, 6.25 nM Hsc70 siRNA or scr. siRNA at the same concentrations (for details see Section 2). (A) Cells were lysed 80 hr after transfection and cell lysates were used for Hsp70 and Hsc70 immunoblots. 16 hr before lysis HT22 cells were pre-incubated for 1 hr at 42 °C to clearly determine Hsp70 knockdown efficiency. The insert of the middle panel shows the blot density of Hsp70 [% of control]. Representative immunoblots of Hsp70 and Hsc70 are shown. Hsp70 and Hsc70 concentrations were related to the density of Ponceau staining. Columns are the means ± SD, n = 4–7, ***P < 0.005 *vs*. control, + + P < 0.01 *vs*. heat shock control, ### *P* < 0.005 *vs*. scr. siRNA, ## *P* < 0.01 *vs*. scr. siRNA, # *P* < 0.05 *vs*. scr. siRNA. B) 80 hr after siRNA transfection, MTT viability assay (see methods) was performed. Columns are the means ± SD, n = 3–4, ****P < 0.001 *vs*. control, ***P < 0.005 *vs*. control, ### *P* < 0.005 *vs*. scr. siRNA, ## *P* < 0.01 *vs*. scr. siRNA.

We found an 80% decrease of Hsp70 expression after 1 hr of heat shock in cells transfected with siRNA against Hsp70, compared to cells transfected with scrambled siRNA or no siRNA (Fig. 3A, left panel). Additionally, Hsc70 expression was 50% lower in cells treated with the siRNA against Hsc70, than in cells treated with scrambled siRNA or no siRNA (Fig. 3A, center panel). While Hsp70 knockdown did not affect the Hsc70 expression levels, Hsc70 knockdown was accompanied by an Hsp70 up-regulation of more than 7000% compared to non-treated cells. Therefore, we treated cells simultaneously with both siRNAs, which resulted in a 50% Hsc70 knockdown and an almost complete block of Hsp70 up-regulation (Fig. 3A, right panel). While Hsp70 knockdown did not affect cell viability, Hsc70 knockdown, both alone or in combination with Hsp70 knockdown, was more toxic and resulted in loss of viability to about 25–30%.

**Fig. 4:**
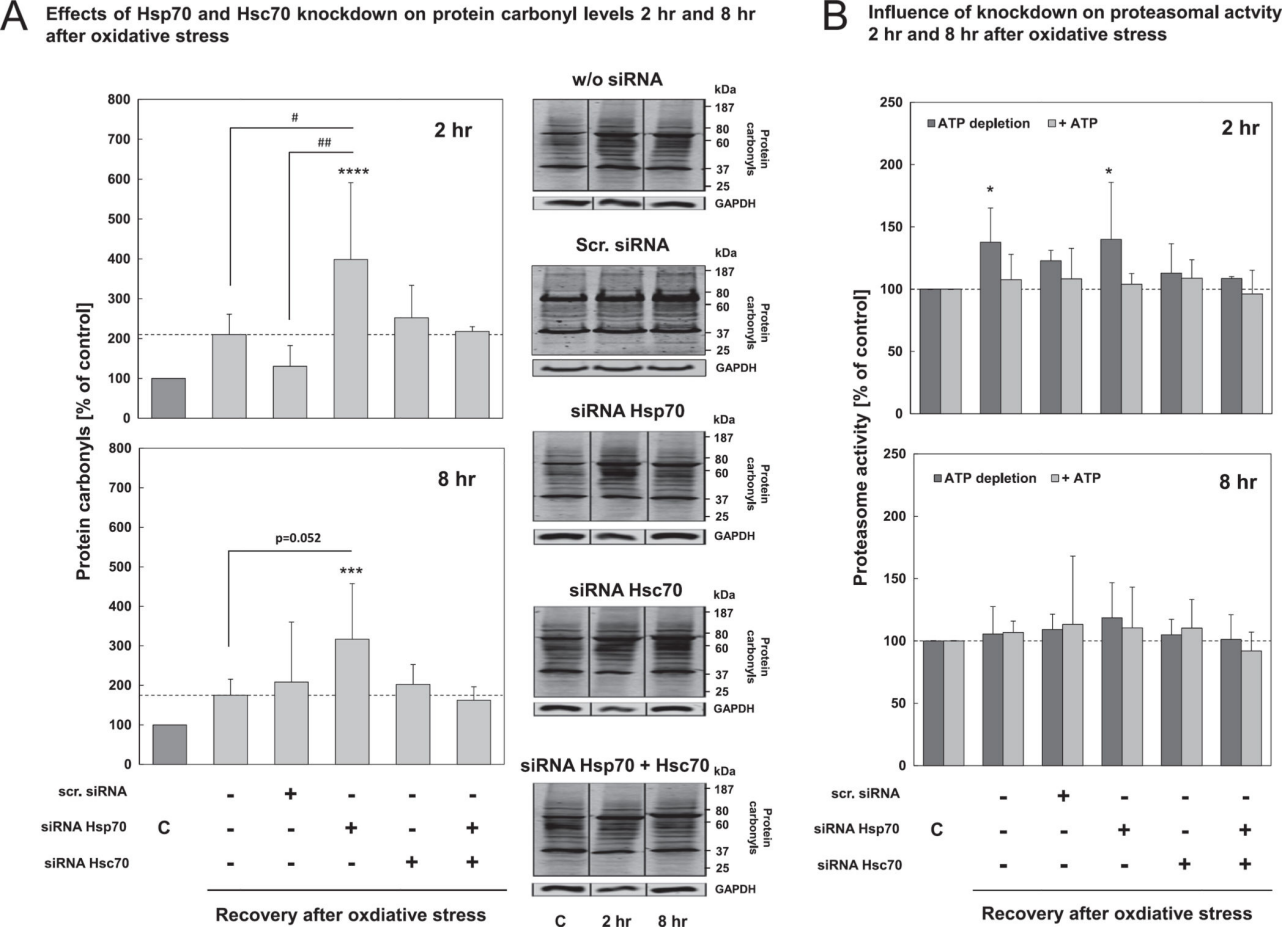
Hsp70 knockdown leads to increased protein carbonyl levels after oxidative stress. HT22 cells were transfected with siRNA against Hsp70 and/or Hsc70. 72 hr after transfection, cells were treated with 0.5 mM hydrogen peroxide for 0.5 hr, control cells were treated with PBS. (A) Cells were lysed 2 hr or 8 hr after hydrogen peroxide treatment and the lysates were used for protein carbonyl immunoblot (see Section 2). Representative immunoblots of protein carbonyls are shown. The two panels of Fig. 3A show the increase in protein carbonyl levels at 2 hr and at 8 hr after oxidative stress compared with the related controls. The columns are the means ± SD, n = 3–6, ***P < 0.005 *vs*. control, ****P < 0.001 *vs*. control, # *P* < 0.05 *vs*. w/o siRNA, ## *P* < 0.01 *vs*. scr. siRNA. (B) Cells were lysed with proteasome activity lysis buffer at 2 hr and 8 hr after hydrogen peroxide treatment, and used for measurement of ATP-dependent (26S) and ATP-independent (20S) proteasomal activity (see methods). Each column reports means ± SD, n = 3–6, *P < 0.05 *vs*. control.

To investigate whether the chaperone knockdown would affect the accumulation of oxidized proteins, we checked for protein carbonyl levels upon recovery after oxidative stress. Compared with cells not treated with siRNA, showing an increase in protein carbonyl levels of 200% 2 hr after hydrogen peroxide treatment, we found that Hsp70 knockdown resulted in considerably higher protein carbonyl levels of almost 400% (Fig. 4A, left panel). Hsc70 knockdown resulted in barely increased protein carbonyl levels of 250%. The effects were also evident after 8 hr of recovery from hydrogen peroxide treatment, albeit at a lesser magnitude (Fig. 4A, right panel). To exclude the possibility that the effect of Hsc70-knockdown is influenced by the accompanying Hsp70 up-regulation, the experiments showed in Fig. 4A were also performed with cells treated with siRNA against both chaperones. As mentioned above, these cells show also a 50% knockdown of Hsc70 with a correspondingly moderate upregulation of Hsp70 (Fig. 3). We found that the double knockdown did not lead to changes in protein carbonyl levels 2 hr and 8 hr after hydrogen peroxide treatment compared to Hsc70 knockdown cells (Fig. 4A). Furthermore, we wanted to exclude the possibility that the increased protein carbonyl levels may be a result of proteasome impairment due to siRNA treatment. Thus, we measured both 20S (ATP-independent) and 26S (ATP-dependent) proteasomal activities in Hsp70, Hsc70 and double knockdown cells, as well as in cells treated with scrambled siRNA. ATP-independent activity was significantly increased 2 hr after oxidative stress in cells not transfected with siRNA. Nevertheless, neither ATP-independent 20S, nor ATP-dependent 26S proteasomal activity were influenced by Hsc70 or Hsp70 siRNA treatment, or by the RNAi experiment in general (Fig. 4B).

### 3.3. Proteasomal degradation of oxidized proteins is enhanced by Hsp70

**Fig. 5:**
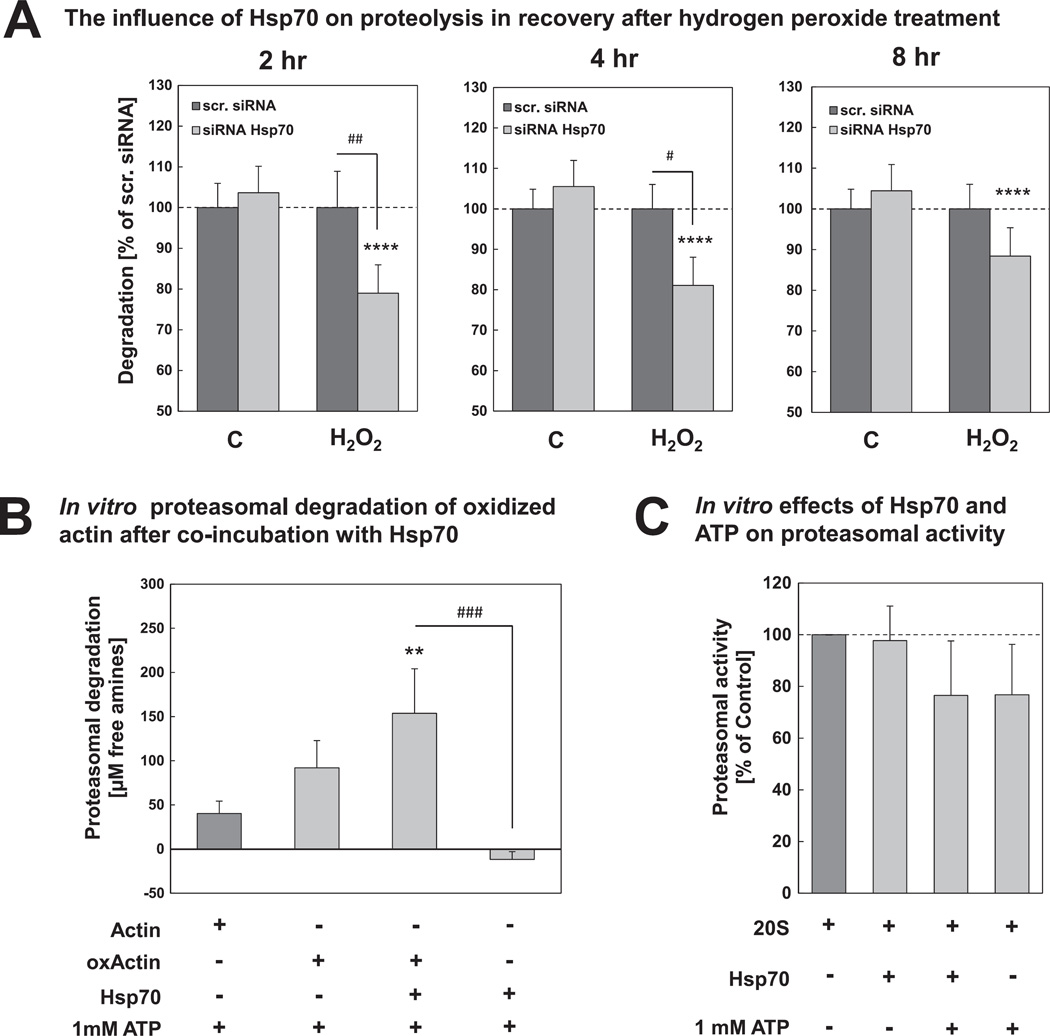
Proteasomal degradation is increased in the presence of Hsp70. (A) HT22 cells were transfected with 25 nM scr. siRNA or 25 nM Hsp70 siRNA 24 hr after seeding. 72 hr after transfection, cells were incubated with growth medium, containing [^35^S]-labeled methionine and cysteine for 2 hr (for details see Section 2), washed and treated with 0.5 mM hydrogen peroxide or PBS for 0.5 hr. Afterwards, growth medium supplemented with 10 mM methionine/cysteine was added. Proteolytic degradation was measured at the desired time points in recovery after oxidative stress, as described in Section 2. The columns represent the proteolytic degradation (in % of scr. siRNA samples) at different time points after oxidative stress. They are the means ± SD, n = 7–9. ****P < 0.001 *vs*. control cells, ## *P* < 0.01 *vs*. scr. siRNA, # *P* < 0.05 *vs*. scr. siRNA. (B) Actin was incubated with hydrogen peroxide for 2 hr at 25 °C. Afterwards, hydrogen peroxide was removed by adding catalase. Oxidized actin (oxActin) was incubated with isolated 20S proteasome and with Hsp70 for 2 hr at 37 °C. Non-oxidized, native actin was used for comparison. Controls for each sample were incubated without the addition of 20S proteasome. The proteasomal degradation was measured using fluorescamine assay (for details see methods). The columns represent the proteasomal degradation, calculated as the difference between proteasome and control samples, respectively. They are the means ± SD, n = 3–4. **P < 0.01 *vs*. actin control, ### *P* < 0.005 *vs*. Hsp70 control. (C) Isolated 20S proteasome was incubated with Hsp70 and/or ATP and proteasomal activity was measured using the fluorogenic proteasome substrate suc-LLVY-MCA as described in methods. Columns are the means ± SD, n = 3.

Our experiments in HT22 cells show that Hsp70 knockdown but not Hsc70 knockdown leads to an accumulation of oxidized proteins, indicating that Hsp70 might actually be involved in some way in the removal of oxidized proteins. Thus, in the next step we wanted to test the influence of Hsp70 on degradation of oxidized proteins in our cell model. Therefore, we labeled short-lived cellular proteins using [^35^S]methionine/cysteine and measured the proteolysis in recovery after oxidative stress in cells, transfected with non-targeting scrambled siRNA or siRNA against Hsp70. As shown in Fig. 5A, Hsp70 knockdown leads to a significant decrease to 80% in the degradation of short-lived proteins 2 hr and 4 hr after hydrogen peroxide treatment compared to cells treated with scr. siRNA. In contrast, Hsp70 knockdown did not affect the proteolysis in control cells, not treated with hydrogen peroxide. 8 hr after oxidative stress Hsp70 knockdown still leads to decreased degradation compared to control cells, however, there is no significant difference compared to scr. siRNA cells also treated with hydrogen peroxide.

In order to establish whether Hsp70 directly influences proteasomal degradation of oxidized proteins we performed additionally an *in vitro* experiment using isolated 20S proteasome, recombinant Hsp70, and both, native actin and oxidized actin as proteasomal substrates. We performed the fluorescamine assay to measure the amount of free amines. Proteasomal degradation was calculated as difference between the sample with 20S proteasome and without 20S proteasome to exclude proteasome-independent effects. We were able to show that oxidized actin is a better substrate for 20S proteasomal degradation than the non-oxidized form, since the amount of free amines is more than doubled compared to non-oxidized actin (Fig. 5A). Interestingly, the addition of Hsp70 to oxidized actin resulted in an even higher formation of free amines from 90 µM to more than 150 µM. Thus, Hsp70 enhances the degradation of oxidized actin. To exclude that this effect may result from the proteasomal degradation of Hsp70, in a further approach, we tested whether Hsp70 is degraded by the 20S proteasome. However, the incubation of the 20S proteasome with Hsp70 did not lead to increased formation of free amines. Moreover, we checked whether there might be a non-specific influence of Hsp70 and/or ATP on maximal 20S proteasomal activity against a non-physiological, fluorogenic peptide substrate, however, the ability of 20S proteasome to degrade the fluorogenic peptide substrate was not changed by either Hsp70 or ATP (Fig. 5B).

### 3.4. Hsp70 interacts with oxidized proteins and with the 20 S proteasome in response to oxidative stress

**Fig. 6:**
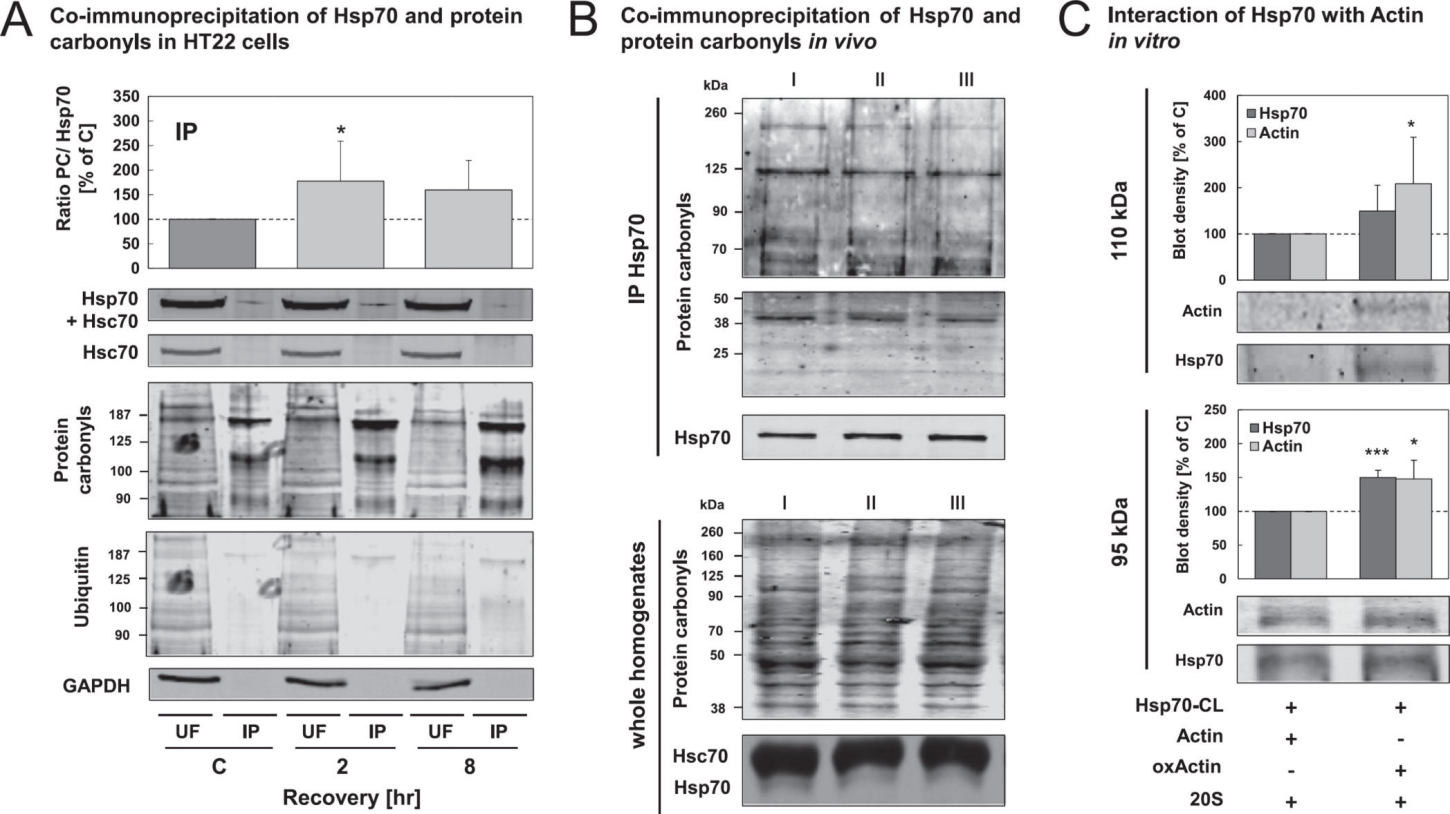
Interaction of Hsp70 with oxidized proteins is increased during recovery from oxidative stress. (A) HT22 cells were incubated with hydrogen peroxide for 0.5 hr. 2 hr and 8 hr after the treatment, cells were lysed using PBS. Hsp70 was immunoprecipitated from the cell lysates (as described in methods). Unbound fractions (UF) and immunoprecipitate (IP) were used for immunoblots against Hsp70, Hsc70, protein carbonyls and ubiquitinated proteins. Representative blots are shown. The blot densities of Hsp70 and protein carbonyls in the IP were quantified. The columns display the ratios between protein carbonyls and Hsp70 (PC/Hsp70) and are the means ± SD, n = 7, *P < 0.05 *vs*. control. (B) Brains from three 18-month old 129/SV mice were homogenized in PBS. Hsp70 was immunoprecipitated from the homogenates. The immunoprecipitates (IP) (upper panel) and the whole homogenates (lower panel) were used for immunoblots against Hsp70, Hsc70 and protein carbonyls. (C) Hsp70, pre-incubated with a heterobifunctional cross-linker (CL) (see methods) was incubated with actin/oxidized actin (oxActin) and the 20S proteasome. After 0.5 hr, the cross-linker was activated *via* UVA exposure for 15 min. Interactions of Hsp70 were analyzed using immunoblots against Hsp70 and actin. Two bands, resulting from cross-linking (95 kDA and 110 kDa) were detected and quantified. Additionally, these bands were analyzed by mass spectrometry (see Table 1). Columns are means ± SD, n = 4–5, *P < 0.05 *vs*. non-oxidized actin, ***P < 0.005 *vs*. non-oxidized actin.

Our results, demonstrating that Hsp70 promotes oxidized protein degradation, further encouraged us to ask whether Hsp70 can mediate a direct interaction between an oxidized protein substrate and the proteasome. To first investigate whether an interaction of Hsp70 with oxidized proteins occurs, we performed immunoprecipitation of Hsp70 from lysates of oxidant-treated cells, using a specific mouse antibody against the inducible Hsp70 (without cross-reactivity to Hsc70) (Fig. 6A). Both the unbound fraction (UF) and the immunoprecipitate (IP) were then analyzed by SDS-PAGE with subsequent immunoblot. Using a rabbit anti-Hsp70/Hsc70 antibody and a specific anti-Hsc70 antibody (a specific rabbit anti-Hsp70 antibody is not available), we were able to demonstrate the immunoprecipitation of anti-Hsp70 solely. The Hsp70 immunoprecipitates were then analyzed for protein carbonyls. To confirm the specificity of the protein carbonyl immunoblots, we performed control experiments (detection without using primary or secondary antibody), which do not show unspecific signals (data not shown). The ratio between protein carbonyl levels and Hsp70 content in the IP was increased to almost 180% 2 hr and 160% 8 hr after oxidant treatment compared to control cells (Fig. 6A). Thus, Hsp70 seems to interact directly with oxidized proteins. Additionally, co-immunoprecipitated proteins did not show a higher ubiquitination in recovery after oxidative stress than did the control cells (Fig. 6A).

As shown in Fig. 6C, an increased interaction of Hsp70 with an oxidized substrate could also be demonstrated *in vitro*. Using a heterobifunctional cross-linker, linked to recombinant Hsp70 in a first step and added to a mixture of actin/oxidized actin and isolated 20S proteasome, we were able to identify two cross-linked products of Hsp70 (72 kDa) and actin (42 kDa), one at nearly 110 kDa and one at 95 kDa, the smaller of which seems to be a cross-linked product of Hsp70 bound to a partially cleaved form of actin. Both cross-linked products are not visible when one of the components is not added to the reaction mixture. With this experiment we were able to demonstrate that the concentration of the 95 kDa actin-Hsp70 interaction product was nearly 1.5-fold higher and the amount of the 110 kDa interaction product even 2-fold higher, when oxidized actin was used instead of non-oxidized actin. Afterwards, the 95 kDa and 110 kDa protein bands were analyzed by MALDI-TOF after in-gel digestion with trypsin to confirm the identity of the proteins. The analysis was performed from five replicates and the results are summarized in Table 1, which reveals that actin and Hsp70 were identified in both protein bands.

To put these results into a greater importance we checked the ability of Hsp70 to bind oxidized proteins also *in vivo*, as shown in Fig. 6B. We found that the interaction between immunoprecipitated Hsp70 and oxidized proteins could also be demonstrated from homogenates of three 18-month old 129/SV mice, indicating that this process occurs also in living organism.

**Fig. 7:**
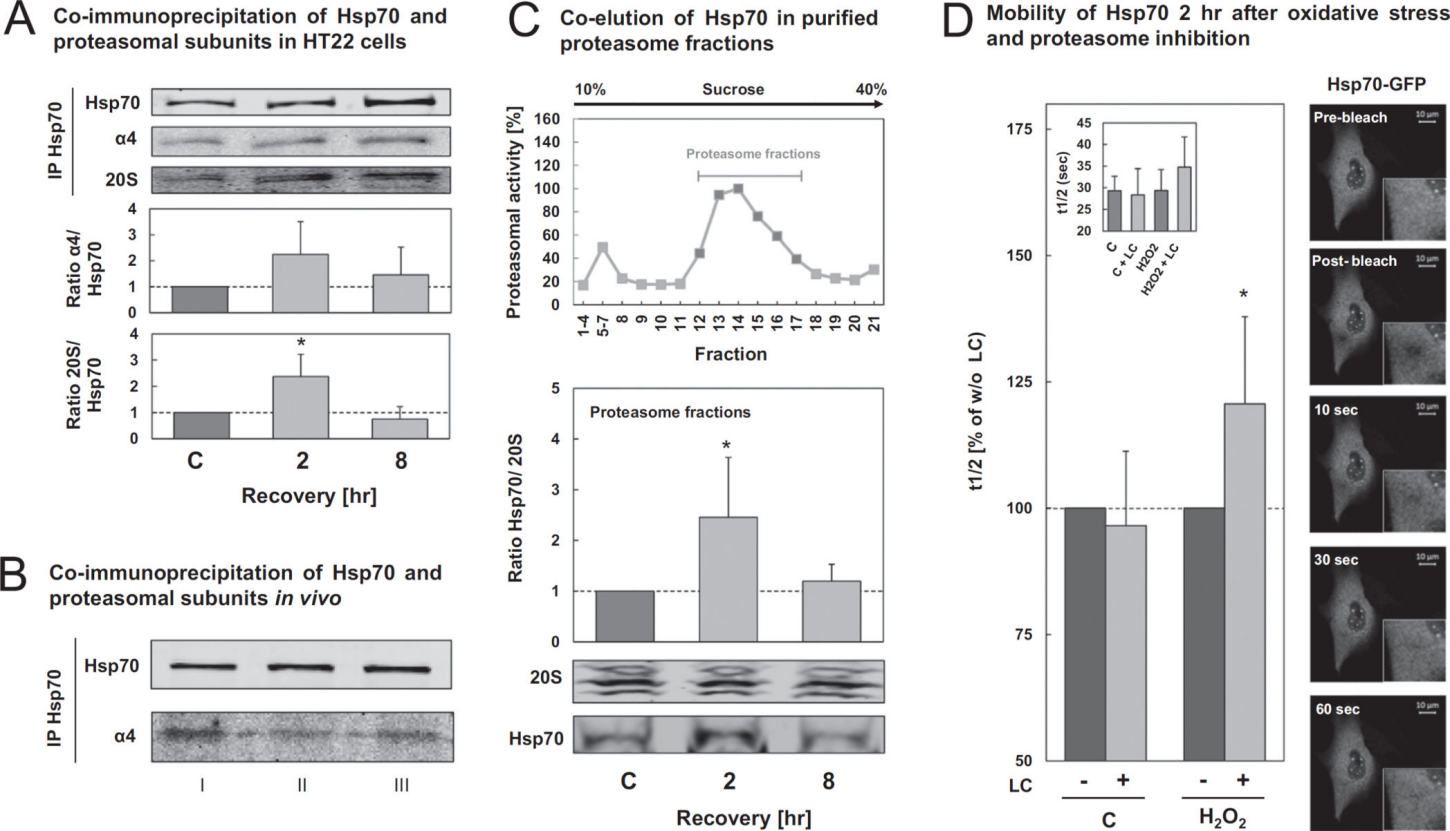
Interaction of Hsp70 with the 20 S proteasome is increased during recovery from oxidative stress. (A) HT22 cells were treated with 0.5 mM hydrogen peroxide for 0.5 hr. After recovery, cells were lysed using PBS. Lysates were used for immunoprecipitation of Hsp70 as described in methods. Immunoprecipitates (IP) were used for immunoblot against Hsp70, proteasomal subunit α4 and 20S core subunits. The columns display the ratios of co-immunoprecipitated proteasomal subunits per immunoprecipitated Hsp70 and are means ± SD, n = 3–4*P < 0.05 *vs*. control. (B) Hsp70 immunoprecipitation was performed from brain homogenates of three 18-month old 129/SV mice. The immunoprecipitates (IP) were used for immunoblots against Hsp70 and the proteasomal subunit α4. In panels C) and D) HT22 cells were transfected with HSPA1A-AC-GFP expression vector 24 hr after seeding. Further 24 hr later cells were incubated with 0.5 mM hydrogen peroxide for 0.5 hr. (C) At the indicated time points after treatment, cells were lysed using PBS, lysates were applied to a 10–40% sucrose gradient. Density gradient centrifugation was performed as described in methods. Fractions were analyzed for proteasomal activity and fractions with a high proteasomal activity were pooled and used for immunoblot against Hsp70 and 20S core subunits. The columns are the ratios between the amount of Hsp70 and the content of proteasomal subunits. The columns are means ± SD, n = 4–5, *P < 0.05 *vs*. control. Panel D) demonstrates the results of FRAP experiments 2 hr after hydrogen peroxide treatment. To inhibit the proteasome, experiments were performed in the presence of 2 µM LC. The data presented show the recovery time t_1/2_ after photobleaching in the presence of LC with the t_1/2_ of the non-LC-treated cells set as 100%. The insert shows the absolute recovery time t_1/2_ of all samples in seconds. The columns are the means ± SD, n = 4–5. *P < 0.05 *vs*. hydrogen peroxide control. Representative fluorescent images of one illustrative FRAP experiment are shown.

Showing that a direct interaction can occur between Hsp70 and oxidized proteins to form a preferred substrate for 20S proteasomal degradation (Figs. 5 and 6), we decided to test whether we could also detect a direct interaction between the 20S proteasome and Hsp70 *in vitro* (HT22 cells) and *in vivo* (129/SV mice). Using two different methods, co-immunoprecipitation and density gradient centrifugation, we were able to demonstrate the interaction of Hsp70 with subunits of the 20S proteasome after oxidative stress in HT22 cells (Fig. 7A+C). We have shown a two-fold increased 20S proteasome co-pull down from Hsp70 immunoprecipitates 2 hr after oxidative stress, compared with control cells. However, 8 hr after oxidative stress the interaction of both proteins declined to basal levels (Fig. 7A). Analogous results were achieved using the density-gradient centrifugation technique (Fig. 7C), where the Hsp70 was co-eluted in the proteasomal fractions from lysates produced 2 hr and 8 hr after hydrogen peroxide exposure. Moreover, as shown in Fig. 7B, the interaction between Hsp70 and the 20S proteasome could also be demonstrated from 18-month old 129/SV murine brain homogenates by performing Hsp70 immunoprecipitation.

To confirm these findings, we performed a “Fluorescence Recovery After Photobleaching” (FRAP) analysis (Fig. 7D). If an interaction of Hsp70 with the 20S proteasome particle occurs, this should influence the dynamics of Hsp70 in the cell. Therefore, we transfected HT22 cells with HSPA1A-GFP vector and treated them with 0.5 mM hydrogen peroxide. In addition, we treated cells with the proteasome inhibitor LC, which should prolong the interaction of the Hsp70-GFP with the 20S proteasome, due to inhibited substrate degradation. A clear increase of the recovery time after photobleaching t_1/2_ could be detected in oxidant-treated cells (2 hr recovery) to almost 125%, from 29s to 35s when co-incubation with LC was performed. In contrast, proteasome inhibition did not change the mobility of Hsp70 in control cells.

## 4. Discussion

The role of Hsp70 in protection against oxidative stress-related damage has been widely accepted [29–31]. However, to our knowledge, a possible function of Hsp70 in promoting the removal of oxidized proteins has not been investigated. In the current study, we are able to demonstrate not only the involvement of Hsp70 in protection against the oxidative stress-related accumulation of oxidized proteins, but also in their proteasomal degradation.

Hsp70 knockdown and prevention of Hsp70 induction during stress resulted in significantly increased levels of protein carbonyls after hydrogen peroxide treatment (Fig. 4A). Although heat shock proteins can refold mildly disordered proteins, it is clear that heat shock proteins are not able to repair covalently-modified oxidized proteins nor to reverse oxidative protein modifications. Thus, we suggested that Hsp70 must somehow be implicated in the removal of oxidized proteins. Moreover, Hsc70 deficiency did not lead to changes in protein carbonyl levels (Fig. 4A) and, therefore, Hsc70 seems not to have a major role in this process. Albeit, Hsp70 and Hsc70 have a quite similar structure, it appears that their participation in (oxidative)-stress induced protein degradation is different. It is postulated that both proteins differ in their C-terminal regions, which may result in different cellular functions [1,32–34]. Hsc70 is an important housekeeping protein [35], mostly responsible for the folding of newly synthesized proteins and involved in maintaining protein homeostasis in non-stressed conditions. In contrast, Hsp70 is mainly responsible for a rapidly inducible cell protection following stress situations [31,36]. Relating to this, we have shown that Hsc70 expression is not affected by oxidative stress, while Hsp70 expression is induced about twofold in our cellular model (Fig. 2), which is comparable to results obtained in other cell lines [37–39]. Since oxidative damage to proteins leads to their unfolding [40,41], the ‘heat shock response’ is activated and the expression of molecular chaperones is increased.

It must be mentioned that Hsp70's ability to prevent accumulation of oxidized proteins (Fig. 4A) may have other reasons besides the assistance in their degradation. On the one hand, it has been shown that Hsp70 is able to protect proteins from severe denaturation caused by different stressors [42,43] since they possess a so-called ‘holdase’ function additionally to their function as ‘foldase’. Heat shock proteins may bind to proteins, thereby stabilizing their structure. Thus, in general Hsp70 and other heat shock proteins have the ability to interact with partially unfolded oxidized proteins, however, since heat shock proteins selectively recognize non-native protein conformations, we suggest that they are not able to prevent the initial oxidation events which lead to the unfolding. On the other hand, it has been shown that especially small heat shock proteins are involved in the formation of large, but less reactive and toxic protein aggregates in aging [44]. Moreover, Gamerdinger et al. as well as Zhang and Qian have suggested that Hsp70 might play a role in sequestration of misfolded proteins to aggresomes when their degradation is inhibited [14,16]. Therefore, we tested whether Hsp70 might prevent accumulation of oxidized proteins following oxidative stress as shown in Fig. 4A by assistance in aggregate formation. However, we were able to demonstrate that Hsp70 knockdown does not lead to changes in aggregate formation (data not shown), indicating that Hsp70 is not involved in this process. Moreover, contrary to this, it has often been shown that Hsp70 and other molecular chaperones are involved in preventing stress-related aggregate formation by binding to denatured proteins, thereby keeping them in a re-foldable or degradable state [42,43,45,46].

If heat shock proteins are not able to refold misfolded or denatured proteins, co-chaperones are involved in navigating heat shock proteins with their bound substrate proteins to degradation. Extensive evidence shows that oxidized proteins are degraded by the 20S proteasome. Thus, we wanted to investigate whether Hsp70 might have an involvement in this process. It is well known that, in contrast to the 26 S proteasome, the 20S proteasome is able to recognize and degrade unfolded proteins in an ATP- and ubiquitin-independent fashion [5–9]. The 20S proteasome recognizes its substrates by their increased surface hydrophobicity [40,41,47]. ‘Hydrophobic patches’ on the surface of proteins result from oxidation-induced charge changes that cause random (partial) unfolding, with exposure of hydrophobic residues that were previously shielded within the interior of proteins. Moreover, it has been shown that the 20S proteasome is significantly more resistant to oxidative stress than the 26S proteasome [48,49]. According to this, we wanted to test whether Hsp70 might be involved in resistance of the 20S proteasome against oxidative inactivation since it was previously reported that another molecular chaperone Hsp90 seems to contribute to the resistance of the 20S proteasome against oxidative damage [21,50]. However, in the present study, we demonstrate that Hsp70 does not affect proteasomal activity after oxidative stress in HT22 cells (Fig. 4B). Therefore, we conclude that Hsp70 does not seem to contribute in protecting the proteasome against oxidative damage, as it was demonstrated for Hsp90.

There is increasing evidence that members of the Hsp70 family may be directly implicated in protein degrading processes *via* shuttling their substrates to the degradation machinery, Cuervo has reported for example that Hsc70 is involved in chaperone-mediated autophagy [15]. Importantly, Hsc70/Hsp70 have been implicated in protein degradation *via* the 26S ubiquitin-proteasome system due to the involvement of the co-chaperones CHIP (carboxyl terminus of Hsc70 interacting protein), which acts as ubiquitin ligase, and BAG1 (BCL2-associated athanogene), which coordinates the binding of Hsp70-substrate-complex to the 26S proteasome [17,19,20,51,52]. Subsequently we wanted to investigate the possibility of a direct influence of Hsp70 on 20S proteasomal degradation of oxidized proteins. Consistent with earlier publications [7,9,53], we found that the oxidized form of a protein, in our case actin, is more efficiently degraded by the 20S proteasome than the non-oxidized native protein (Fig. 5B). Furthermore, the addition of Hsp70 resulted in a significantly higher increase in 20S proteasomal degradation of oxidized actin (Fig. 5B). Moreover, we were able to confirm this in our cell model, where we demonstrated that Hsp70 knockdown affects the degradation of oxidized proteins especially in the early phase after oxidative stress (Fig. 5A). Consequently, we suggest a direct involvement of Hsp70 in the degradation process during, and in recovery after, oxidative stress.

Whittier et al. have shown that Hsp90 is able to bind oxidized Calmodulin and promote its degradation by the 20S proteasome by mediating their interaction [22]. According to this, our results encouraged us to investigate the interaction of Hsp70 with oxidized proteins and the 20S proteasome. Callahan et al. [34] showed the ability of Hsp70 and Hsc70 to bind oxidized peptides. Moreover, they demonstrated that Hsp70 is a better interaction partner for oxidized peptides than is Hsc70, supporting our findings that Hsp70 may play the more important role in the removal of oxidized proteins (Fig. 4A). The reason for this difference between both chaperones, apparently, is a small variance in the C-terminus of the two chaperones, which forms the substrate-binding site. Callahan et al. demonstrated that oxidative conditions lead to conformational changes which enhance the peptide accessibility to the peptide-binding-pocket. Lazarev et al. found that Hsp70 binds to oxidized GAPDH, preventing it from further damage in C6 rat glioblastoma cells [54]. We were able to confirm these data by demonstrating the ability of Hsp70 to bind oxidized proteins *in vitro,* as well as in our cell model and *in vivo* (Fig. 6). Hsp70 bound to a cross-linker showed a high reactivity to interact with oxidized actin but not with the non-oxidized protein (Fig. 6C). Immunoprecipitation of Hsp70 from 18 month old 129/SV murine brain homogenates showed a co-pull down of oxidized proteins (Fig. 6B), This experiment was also performed with HT22 lysates, resulting in an increased pull-down of oxidized proteins to about 180% 2 hr after hydrogen peroxide treatment (Fig. 6A). Interestingly, these oxidized proteins bound to Hsp70 did not show a higher polyubiquitination, which further supports the widely accepted assumption that oxidized proteins are degraded by the 20S proteasome in an ubiquitin-independent way. It has been demonstrated that oxidized proteins are not preferentially ubiquitinated and that an intact ubiquitination system is not required for their degradation [5–9].

Using various techniques, such as co-immunoprecipitation, and co-elution of Hsp70 in density gradient centrifugation purified proteasome fractions and measurement of Hsp70 mobility after proteasome inhibition by FRAP, we demonstrated that Hsp70 interacts additionally with the 20S proteasome (Fig. 7), confirming our hypothesis that Hsp70 seems to mediate the interaction between oxidized proteins and the 20S proteasome. Although, we suggest that Hsp70 is not involved in modulating 20S proteasome integrity after oxidative stress as discussed above (Fig. 4B), we have shown that ATP-independent activity, which correlates with the activity of the 20S proteasome, is increased 2 hr after oxidative stress (Fig. 4B). It is known that the activity of the 20S proteasome is regulated by various redox-regulated post-translational modifications, such as S-glutathionylation. It is assumed that S-glutathionylation of some 20S proteasome α-subunits stabilizes the open conformation of the 20S proteasome, which is normally closed when regulators such as 19S or 11S are not bound, thereby stimulating the substrate entrance into the catalytic core [55,56]. Moreover, phosphorylation of the α-subunits *via* the polo-like kinase has been shown to activate the 20S proteasome [57]. These redox-regulated post-translational modifications may be essential for Hsp70's ability to mediate the interaction between the oxidized bound substrate with the α-subunits of the 20S proteasome, promoting their fast entry into the catalytic core and their degradation.

**Fig. 8:**
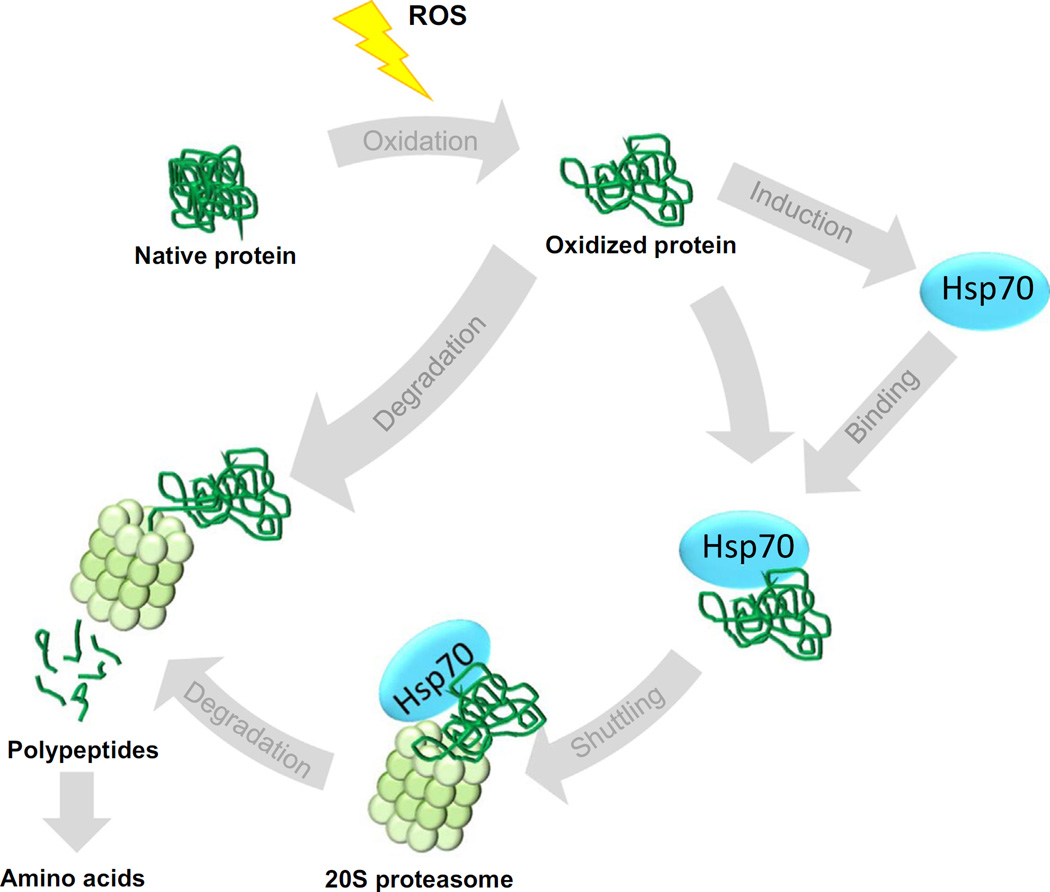
Scheme of the proposed role of Hsp70 in proteasomal degradation of oxidized proteins. Various reactive species (ROS) can oxidize native, functional and properly folded proteins. Oxidized proteins are degraded by the 20S proteasome, which recognizes oxidized proteins *via* their partial unfolding and, therefore, increased surface hydrophobicity. The oxidized proteins are degraded to peptides and small polypeptides, which are further cleaved to amino acids. These amino acids are then available for new protein synthesis, demonstrating the importance of proteasomal degradation in maintenance of an amino acid pool for biosynthesis of proteins during oxidative stress. As a result of stress-related protein unfolding, Hsp70 expression is increased. Hsp70 is also able to recognize and bind oxidized proteins due to their increased surface hydrophobicity. Hsp70 then shuttles the oxidized protein to the 20S proteasome. This facilitates the rapid and selective degradation of oxidized proteins and, therefore, is suggested to minimize the interactions of oxidized proteins with other proteins, and the consequent formation of protein aggregates.

Taking together, the results presented in our current study demonstrate the involvement of the stress-inducible molecular chaperone Hsp70 in the 20S proteasomal degradation of oxidized proteins. As shown, in Fig. 8, we suggest that in the early phase after oxidative stress, Hsp70 binds to partially unfolded oxidized proteins and keeps them in a soluble, degradable form. Oxidized proteins bound to Hsp70 can then migrate to 20S proteasomes where they can be efficiently degraded. Thus, besides the direct recognition of oxidized protein substrates by the 20S proteasome, there seems to be another, Hsp70-mediated, way to catalyze the efficient degradation of oxidized proteins.

Future studies should investigate the involvement of co-chaperones/interacting proteins and co-factors which may be involved in this process and which modulate the ability of Hsp70 to mediate shuttling of oxidized proteins to the 20S proteasome. Moreover possible interaction sites of Hsp70 on 20S proteasome subunits remain to be identified. It is assumed that the molecular chaperone Hsp90 is able to bind directly to the α-subunits of the 20S proteasome [22,58]. However, in contrast, it is suggested that the co-chaperone BAG1 is mediating the interaction between the 26S proteasome and Hsc70/Hsp70 [19,52]. Understanding of Hsp70's ability to promote the degradation of oxidatively damaged proteins may be especially important in the field of aging research, since it is known that oxidized proteins and protein aggregates accumulate during aging and various age-related diseases due to the impairment of the proteasomal system [59,60]. Furthermore, there is increasing evidence that the stress-related inducibility of Hsp70 expression declines in aged cell models and organisms [61–64] and that the chaperones are overloaded in aged cells due to increasing formation and accumulation of oxidized proteins [65,66]. Thus, modulating Hsp70 levels may be a possible pharmaceutical goal to maintain protein homeostasis and prevent the formation of toxic protein aggregates that can disrupt cellular function.

## Figures and Tables

**Table 1:** Proteins identified in cross-linking experiments.

kDa	Protein name (gene name)	Accession number	Mascot score	% MS coverage	% sequence coverage
**95**	Heat shock 70 kDa protein 1A (HSPA1A)	P0DMV8	156	38.3	53.3
	Actin (ACTB)	P60709	61	17.0	30.9
**110**	Heat shock 70 kDa protein 1A (HSPA1A)	P0DMV8	141	42.6	50.5
	Actin (ACTB)	P60709	86	29.4	60.8

## Abbreviations

AMC: 7-amino-4-methylcoumarin
CL: Cross-linker
DNP: Dinitrophenyl
FRAP: Fluorescence recovery after photobleaching
GFP: Green fluorescent protein
Hsc70: Heat shock cognate protein 70 (also referred to as Hsp73)
Hsp70: Heat shock protein 70 (also referred to as Hsp72 or Hsp70-1)
IP: Immunoprecipitate
LC: Lactacystin
oxActin: Oxidized actin
PC: Protein carbonyl
Scr: Scrambled
UF: Unbound Fraction

## References

[1] Daugaard M, Rohde M, Jaattela M (2007). The heat shock protein 70 family: Highly homologous proteins with overlapping and distinct functions. FEBS Lett.

[2] Castro JP, Ott C, Jung T, Grune T, Almeida H (2012). Carbonylation of the cytoskeletal protein actin leads to aggregate formation. Free Radic. Biol. Med.

[3] Hohn A, Jung T, Grimm S, Grune T (2010). Lipofuscin-bound iron is a major intracellular source of oxidants: role in senescent cells. Free Radic. Biol. Med.

[4] Anckar J, Sistonen L (2011). Regulation of HSF1 function in the heat stress response: implications in aging and disease. Annu. Rev. Biochem.

[5] Grune T, Reinheckel T, Davies KJ (1997). Degradation of oxidized proteins in mammalian cells. FASEB J.

[6] Grune T, Reinheckel T, Joshi M, Davies KJ (1995). Proteolysis in cultured liver epithelial cells during oxidative stress. Role of the multicatalytic proteinase complex, proteasome. J. Biol. Chem.

[7] Shringarpure R, Grune T, Mehlhase J, Davies KJ (2003). Ubiquitin conjugation is not required for the degradation of oxidized proteins by proteasome. J. Biol. Chem.

[8] Kastle M, Grune T (2011). Proteins bearing oxidation-induced carbonyl groups are not preferentially ubiquitinated. Biochimie.

[9] Davies KJ (2001). Degradation of oxidized proteins by the 20S proteasome. Biochimie.

[10] Goldberg AL (2003). Protein degradation and protection against misfolded or damaged proteins. Nature.

[11] Lecker SH, Goldberg AL, Mitch WE (2006). Protein degradation by the ubiquitin-proteasome pathway in normal and disease states. J. Am. Soc. Nephrol.

[12] Naujokat C, Hoffmann S (2002). Role and function of the 26S proteasome in proliferation and apoptosis. Lab. Investig. J. Tech. Methods Pathol.

[13] Grune T, Catalgol B, Licht A, Ermak G, Pickering AM, Ngo JK (2011). HSP70 mediates dissociation and reassociation of the 26S proteasome during adaptation to oxidative stress. Free Radic. Biol. Med.

[14] Zhang X, Qian SB (2011). Chaperone-mediated hierarchical control in targeting misfolded proteins to aggresomes. Mol. Biol. Cell.

[15] Cuervo AM (2010). Chaperone-mediated autophagy: selectivity pays off. Trends Endocrinol. Metab.

[16] Gamerdinger M, Kaya AM, Wolfrum U, Clement AM, Behl C (2011). BAG3 mediates chaperone-based aggresome-targeting and selective autophagy of misfolded proteins. EMBO Rep.

[17] Kundrat L, Regan L (2010). Balance between folding and degradation for Hsp90-dependent client proteins: a key role for CHIP. Biochemistry.

[18] Park SH, Bolender N, Eisele F, Kostova Z, Takeuchi J, Coffino P (2007). The cytoplasmic Hsp70 chaperone machinery subjects misfolded and endoplasmic reticulum import-incompetent proteins to degradation via the ubiquitin-proteasome system. Mol. Biol. Cell.

[19] Luders J, Demand J, Hohfeld J (2000). The ubiquitin-related BAG-1 provides a link between the molecular chaperones Hsc70/Hsp70 and the proteasome. J. Biol. Chem.

[20] Muller P, Ruckova E, Halada P, Coates PJ, Hrstka R, Lane DP (2013). C-terminal phosphorylation of Hsp70 and Hsp90 regulates alternate binding to co-chaperones CHIP and HOP to determine cellular protein folding/degradation balances. Oncogene.

[21] Conconi M, Petropoulos I, Emod I, Turlin E, Biville F, Friguet B (1998). Protection from oxidative inactivation of the 20S proteasome by heat-shock protein 90. Biochem. J.

[22] Whittier JE, Xiong Y, Rechsteiner MC, Squier TC (2004). Hsp90 enhances degradation of oxidized calmodulin by the 20 S proteasome. J. Biol. Chem.

[23] Catalgol B, Wendt B, Grimm S, Breusing N, Ozer NK, Grune T (2010). Chromatin repair after oxidative stress: role of PARP-mediated proteasome activation. Free Radic. Biol. Med.

[24] Dalle-Donne I, Rossi R, Giustarini D, Milzani A, Colombo R (2003). Protein carbonyl groups as biomarkers of oxidative stress. Clin Chim Acta.

[25] Hough R, Pratt G, Rechsteiner M (1987). Purification of two high molecular weight proteases from rabbit reticulocyte lysate. J. Biol. Chem.

[26] Hohn A, Jung T, Grimm S, Catalgol B, Weber D, Grune T (2011). Lipofuscin inhibits the proteasome by binding to surface motifs. Free Radic. Biol. Med.

[27] Catalgol B, Grune T (2009). Turnover of oxidatively modified proteins: the usage of in vitro and metabolic labeling. Free Radic. Biol. Med.

[28] Candiano G, Bruschi M, Musante L, Santucci L, Ghiggeri GM, Carnemolla B (2004). Blue silver: a very sensitive colloidal Coomassie G-250 staining for proteome analysis. Electrophoresis.

[29] Subrizi A, Toropainen E, Ramsay E, Airaksinen AJ, Kaarniranta K, Urtti A (2015). Oxidative stress protection by exogenous delivery of rhHsp70 chaperone to the retinal pigment epithelium (RPE), a possible therapeutic strategy against RPE degeneration. Pharm. Res.

[30] Mosser DD, Caron AW, Bourget L, Denis-Larose C, Massie B (1997). Role of the human heat shock protein hsp70 in protection against stress-induced apoptosis. Mol. Cell. Biol.

[31] Mosser DD, Caron AW, Bourget L, Meriin AB, Sherman MY, Morimoto RI (2000). The chaperone function of hsp70 is required for protection against stress-induced apoptosis. Mol. Cell. Biol.

[32] Goldfarb SB, Kashlan OB, Watkins JN, Suaud L, Yan W, Kleyman TR (2006). Differential effects of Hsc70 and Hsp70 on the intracellular trafficking and functional expression of epithelial sodium channels. Proc. Natl. Acad. Sci. USA.

[33] Menoret A, Chaillot D, Callahan M, Jacquin C (2002). Hsp70, an immunological actor playing with the intracellular self under oxidative stress. Int. J. Hyperth.

[34] Callahan MK, Chaillot D, Jacquin C, Clark PR, Menoret A (2002). Differential acquisition of antigenic peptides by Hsp70 and Hsc70 under oxidative conditions. J. Biol. Chem.

[35] Liu T, Daniels CK, Cao S (2012). Comprehensive review on the HSC70 functions, interactions with related molecules and involvement in clinical diseases and therapeutic potential. Pharmacol. Ther.

[36] Kalmar B, Greensmith L (2009). Induction of heat shock proteins for protection against oxidative stress. Adv. Drug Deliv. Rev.

[37] Bianchi A, Moulin D, Hupont S, Koufany M, Netter P, Reboul P (2014). Oxidative stress-induced expression of HSP70 contributes to the inhibitory effect of 15D-PGJ2 on inducible prostaglandin pathway in chondrocytes. Free Radic. Biol. Med.

[38] Lee JS, Jung JH, Kim TH, Seo JS (2004). Changes of Gene Expression in NIH3T3 Cells Exposed to Osmotic and Oxidative Stresses Genomics & Informatics.

[39] Hall L, Martinus RD (2013). Hyperglycaemia and oxidative stress upregulate HSP60 & HSP70 expression in HeLa cells. Springer Plus.

[40] Pacifici RE, Kono Y, Davies KJ (1993). Hydrophobicity as the signal for selective degradation of hydroxyl radical-modified hemoglobin by the multicatalytic proteinase complex, proteasome. J. Biol. Chem.

[41] Giulivi C, Pacifici RE, Davies KJ (1994). Exposure of hydrophobic moieties promotes the selective degradation of hydrogen peroxide-modified hemoglobin by the multicatalytic proteinase complex, proteasome. Arch. Biochem. Biophys.

[42] Hageman J, Vos MJ, van Waarde MA, Kampinga HH (2007). Comparison of intraorganellar chaperone capacity for dealing with stress-induced protein unfolding. J. Biol. Chem.

[43] Michels AA, Kanon B, Konings AW, Ohtsuka K, Bensaude O, Kampinga HH (1997). Hsp70 and Hsp40 chaperone activities in the cytoplasm and the nucleus of mammalian cells. J. Biol. Chem.

[44] Walther DM, Kasturi P, Zheng M, Pinkert S, Vecchi G, Ciryam P (2015). Widespread proteome remodeling and aggregation in aging *C. elegans*. Cell.

[45] Kampinga HH, Brunsting JF, Stege GJ, Burgman PW, Konings AW (1995). Thermal protein denaturation and protein aggregation in cells made thermotolerant by various chemicals: role of heat shock proteins. Exp. Cell Res.

[46] Daturpalli S, Waudby CA, Meehan S, Jackson SE (2013). Hsp90 inhibits alpha-synuclein aggregation by interacting with soluble oligomers. J. Mol. Biol.

[47] Lasch P, Petras T, Ullrich O, Backmann J, Naumann D, Grune T (2001). Hydrogen peroxide-induced structural alterations of RNAse A. J. Biol. Chem.

[48] Reinheckel T, Sitte N, Ullrich O, Kuckelkorn U, Davies KJ, Grune T (1998). Comparative resistance of the 20S and 26S proteasome to oxidative stress. Biochem. J.

[49] Reinheckel T, Ullrich O, Sitte N, Grune T (2000). Differential impairment of 20S and 26S proteasome activities in human hematopoietic K562 cells during oxidative stress. Arch. Biochem. Biophys.

[50] Conconi M, Szweda LI, Levine RL, Stadtman ER, Friguet B (1996). Age-related decline of rat liver multicatalytic proteinase activity and protection from oxidative inactivation by heat-shock protein 90. Arch. Biochem. Biophys.

[51] Murata S, Minami Y, Minami M, Chiba T, Tanaka K (2001). CHIP is a chaperone-dependent E3 ligase that ubiquitylates unfolded protein. EMBO Rep.

[52] Alberti S, Demand J, Esser C, Emmerich N, Schild H, Hohfeld J (2002). Ubiquitylation of BAG-1 suggests a novel regulatory mechanism during the sorting of chaperone substrates to the proteasome. J. Biol. Chem.

[53] Grune T, Blasig IE, Sitte N, Roloff B, Haseloff R, Davies KJ (1998). Peroxynitrite increases the degradation of aconitase and other cellular proteins by proteasome. J. Biol. Chem.

[54] Lazarev VF, Nikotina AD, Mikhaylova ER, Nudler E, Polonik SG, Guzhova IV (2016). Hsp70 chaperone rescues C6 rat glioblastoma cells from oxidative stress by sequestration of aggregating GAPDH. Biochem. Biophys. Res. Commun.

[55] Demasi M, Netto LE, Silva GM, Hand A, de Oliveira CL, Bicev RN (2014). Redox regulation of the proteasome via S-glutathionylation. Redox Biol.

[56] Silva GM, Netto LE, Simoes V, Santos LF, Gozzo FC, Demasi MA (2012). Redox control of 20S proteasome gating. Antioxid. Redox Signal.

[57] Feng Y, Longo DL, Ferris DK (2001). Polo-like kinase interacts with proteasomes and regulates their activity. Cell Growth Differ.

[58] Realini C, Rogers SW, Rechsteiner M (1994). KEKE motifs. Proposed roles in protein-protein association and presentation of peptides by MHC class I receptors. FEBS Lett.

[59] Davies KJ, Shringarpure R (2006). Preferential degradation of oxidized proteins by the 20S proteasome may be inhibited in aging and in inflammatory neuromuscular diseases. Neurology.

[60] Reeg S, Grune T (2015). Protein oxidation in aging: does it play a role in aging progression?. Antioxid. Redox Signal.

[61] Schumpert C, Handy I, Dudycha JL, Patel RC (2014). Relationship between heat shock protein 70 expression and life span in Daphnia. Mech. Ageing Dev.

[62] Heydari AR, Wu B, Takahashi R, Strong R, Richardson A (1993). Expression of heat shock protein 70 is altered by age and diet at the level of transcription. Mol. Cell. Biol.

[63] Fargnoli J, Kunisada T, Fornace AJ, Schneider EL, Holbrook NJ (1990). Decreased expression of heat shock protein 70 mRNA and protein after heat treatment in cells of aged rats. Proc. Natl. Acad. Sci. USA.

[64] Ambra R, Mocchegiani E, Giacconi R, Canali R, Rinna A, Malavolta M (2004). Characterization of the hsp70 response in lymphoblasts from aged and centenarian subjects and differential effects of in vitro zinc supplementation. Exp. Gerontol.

[65] Soti C, Csermely P (2003). Aging and molecular chaperones. Exp. Gerontol.

[66] Proctor CJ, Soti C, Boys RJ, Gillespie CS, Shanley DP, Wilkinson DJ (2005). Modelling the actions of chaperones and their role in ageing. Mech. Ageing Dev.

